# Effects of white matter hyperintensities on isolated executive function assessed by the Trail Making Test

**DOI:** 10.12688/f1000research.139557.1

**Published:** 2023-08-22

**Authors:** Tomiko Nagayama, Shuhei Yamaguchi, Takuya Nakayama, Sunghoon Yang, Seiichi Inagaki, Masao Nagayama

**Affiliations:** 1 Neurology, International University of Health and Welfare,Atami Hospital, Atami, Shizuoka, 413-0012, Japan; 2 Shimane Prefecture Bureau of Hospital Administration, Shimane Prefectural Center Hospital, Izumo, Shimane, 693-8555, Japan; 3 Rehabilitation, International University of Health and Welfare, Atami Hospital, Atami, Shizuoka, 413-0012, Japan; 4 Neurology, International University of Health and Welfare, Atami Hospital, Atami, Shizuoka, 413-0012, Japan; 5 Graduate School of Public Health, International University of Health and Welfare, Narita, Tiba, 286-8686, Japan; 6 Neurology, International University of Health and Welfare, Atami Hospital, Atami, Shizuoka, 413-0012, Japan

**Keywords:** cerebral white matter hyperintensities, brain MRI, Trail Making Test, cognitive function, age adjustment

## Abstract

Background: White matter hyperintensities (WMHs) on MRI are associated with cognitive dysfunction, particularly slow processing speed and executive dysfunction. However, it is not clear whether WMHs burden affects isolated executive function independent of aging when WMHs are assessed separately in periventricular hyperintensity (PVH) and deep and subcortical white matter hyperintensity (DSWMH).

Purpose: To assess the relationship between the degree of WMHs and the performance on the Trail Making Test (TMT), which can evaluate isolated ability of set-shifting and working memory.

Methods: 74 participants who visited our memory clinic and underwent the TMT subtests (TMT-A and TMT-B) and the Mini-Mental State Examination (MMSE). All subjects performed the TMT within the time limits and their MMSE scores were 24 or higher, and they were diagnosed as having normal cognition or mild cognitive impairment. The extent of PVH and DSWMH was graded from 0 to 3 using the Fazekas scale. We obtained testing time to complete the TMT-A and TMT-B, and calculated TMT-B minus TMT-A. We performed correlation analyses between the degree of WMHs and the time measures of the TMT subtests with adjustment of age.

Results: Average scores of the MMSE were not different among the groups either by PVH grade or by DSWMH grade. In contrast, average time required for the TMT-A, TMT-B, and TMT-B minus TMT-A increased along with exacerbation of PVH and DSWMH grade. After the adjustment of age we found significant association between only DSWMH grade and the time difference of TMT-B minus TMT-A.

Conclusions: Exacerbation of PVH and DSWMH differentially affected isolated executive functions assessed by the TMT subtests independent of age and general cognitive function.

## Introduction

Cerebral white matter hyperintensities (WMHs) appear as hyperintense areas on T2-weighted magnetic resonance image (MRI) and increase with aging. ^1^^,^^2^ While WMHs are often seen in healthy elderlies, they are commonly found in patients with brain diseases such as stroke and dementia. The spread of WMHs is associated with progression of cognitive impairment, ^3^^,^^4^ although the significance of WMHs on different cognitive domains still remains under discussion. Because the forms of WMHs are variable, WMHs are divided into two broad categories depending on the difference in their locations, one is periventricular hyperintensity (PVH) and the other is deep subcortical white matter hyperintensity (DSWMH). The functional significance of these distinct lesions on cognitive functions are also unclear.

Recent studies have revealed that WMHs predominantly affect attention ^5^ and executive function ^6^ rather than general cognitive function or memory function. The Trail Making Test (TMT) was developed as a part of the army individual test battery, ^7^ and has been used for the assessment of visual scanning, spatial attention, psychomotor speed, and executive function. The TMT consists of two parts (TMT-A and TMT-B). The time required to complete each part of the TMT and the time difference (TMT-B minus TMT-A) are used for quantitative measures, which increase with age. In clinical practice the TMT is used as an indicator of neuropsychological impairment associated with various diseases of central nervous system. However, there has been controversy regarding the relationship between distribution and severity of WMHs and measures of the TMT performance.

In this study we investigated the relationship between the degree of PVH and DSWMH and the performance on the TMT and the Mini-Mental State Examination (MMSE) in a clinical population with mild cognitive impairment (MCI) or without cognitive impairment.

## Methods

### Subjects

A total of 74 subjects (55 women), who visited the department of neurology in the Atami hospital in Shizuoka with a chief complaint of forgetfulness or memory disturbance between July 2015 and August 2022, participated in this study. All participants agreed to perform brain MRI scans and cognitive function tests including the MMSE and TMT. The inclusion criterion for the study was that they performed the TMT within the time limits (i.e. 180 sec for the TMT-A and 300 sec for the TMT-B) and their MMSE score was 24 points or higher. Their clinical diagnoses were as follows: 35 subjects without objective cognitive impairment and 39 subjects with MCI. MCI was diagnosed according to the criteria proposed by Petersen. ^8^ The age of the patients ranged from 51 to 89 years old, mean 75.5±6.5 years old.

### Statement of ethics

Patient data were de-identified, and therefore written informed consent was not required. Moreover, information on this study, including the purpose of the study, was published on our hospital’s homepage to guarantee subjects the opportunity to refuse to participate in the study. The study was approved, and this consent protocol was reviewed and the need for written and informed consent was waived by the International University of Health and Welfare Atami Hospital’s Ethics Committee, Shizuoka, Japan (Authorization No.21-A-191).

### Imaging data

We used a 1.5T-MRI scanner (Excelart MRI, Canon, Japan) from July 2015 to July 2018, and a 3T-MRI scanner (Vantage Galan MRI, Canon, Japan) after August 2018. T1-weighted image, T2-weighted image, fluid-attenuated inversion recovery (FLAIR) image, and T2*-weighted image were obtained. For WMHs evaluation we used FLAIR images.

We divided WMHs into two categories: PVH that is symmetrical and adjacent to the lateral ventricles, and DSWMHs that is asymmetrical and apart from the ventricles. According to the Fazekas classification scale, ^9^ PVH was graded from 0 to III; grade 0: absent, grade I: “cap” or pencil-thin lining, grade II: smooth “halo”, and grade III: irregular PVH extending into the deep white matter. DSWMH was also graded from 0 to III; grade 0: absent, grade I: punctate foci (maximum size, <3 mm), grade II: beginning confluence of foci (the size was 3 mm or more), and grade III: large confluent areas. If the left and right cerebral hemispheres showed different grades, the higher one was adopted. The grade was determined based on the consensus of three different neurologists.

### Neuropsychological examinations

The TMT and MMSE were administered to all participants by the rehabilitation staffs. We used the Japanese version of the TMT and measured the time to complete the TMT-A and TMT-B separately. The Japan Society for Higher Brain Dysfunction advocated the criteria for clinical use of the TMT. The criteria for clinical judgment include the testing time required for completion of the TMT and the number of false responses, which were stratified according to age ^10^ ( Table 1). The time limit for completion was 180 s for the TMT-A and 300 s for the TMT-B according to the TMT manual. We judged the results using these criteria for the TMT performance as normal, borderline, or abnormal. We also calculated the time difference of TMT-B minus TMT-A as an index of isolated executive function (i.e. set-shifting and working memory).

**Table 1.** The clinical judgment criteria for the TMT subtests advocated by the Japan Society for Higher Brain Dysfunction.

A) Standard testing time (sec) required for the TMT subtest completion				
Age	Testing time for the TMT-A		Testing time for the TMT-B	
	Mean	SD	Mean	SD
50s	34	6	60	14
60s	35	7	62	13
70s	46	11	88	25
80s	51	9	104	24

B) Criteria for the clinical judgment in the TMT-A			
Number of false responses	Testing time		
	Within mean + 1SD	Between mean + 1SD and 2SD	Longer than mean + 2SD
2 or less	Normal	Borderline	Abnormal
3 or more	Borderline	Abnormal	Abnormal

C) Criteria for the clinical judgment in the TMT-B			
Number of false responses	Testing time		
	Within mean + 1SD	Between mean + 1SD and 2SD	Longer than mean + 2SD
4 or less	Normal	Borderline	Abnormal
5 or more	Borderline	Abnormal	Abnormal

A) shows standard testing time (mean and standard deviation (SD)) required for the TMT subtest completion in each age group. B) and C) show the criteria for the final judgement for the TMT-A and TMT-B, respectively, by combining the time required for task completion and the number of false responses, based on the judgment criteria advocated by the Japan Society for Higher Brain Dysfunction.

### Statistical analysis

We conducted a one-way analysis of variance test without and with age adjustment (ANOVA and ANCOVA) to determine significant differences of continuous variables between the groups with Bonferroni post-hoc comparisons. The chi-square tests were used to determine significant differences of the categorical frequencies between the groups, and also of each ratio of clinical judgement for the TMT-A and the TMT-B. All statistical analyses were performed using SPSS version 23 (IBM Corporation). A value of p < 0.05 was considered statistically significant.

Gender differences were not taken into account in our study design because the number of male patients was smaller than that of female patients, and so the subjects number for each grade of cerebral white matter lesions in the male patients group ranged from 2 to 10, which made the statistical analysis difficult. Also, the TMT is generally reported to have no gender difference. ^11^^,^^12^ Furthermore, the standard criteria for clinical judgment which we used describe the testing time required for completion of the TMT only by age, but not by gender ( Table 1). As a reference, the gender differences results were disclosed in the extended data of the repository.

## Results

The demographic data and the performance data in the neuropsychological tests for each grade of PVH and DSWMH are shown in Table 2. There was no subject who showed grade 0 of PVH or grade 0 of DSWMH. The mean age tended to increase as the grade of PVH and DSWMH increased (The DSWMH grade I vs. III groups: p=0.045). The prevalence of hypertension and dyslipidemia increased in the groups of DSWMH grade II and III compared to the grade I group (the grade I vs. II groups: p=0.003, grade I vs. III groups: p=0.027 for the prevalence of hypertension). The education periods were comparable among all groups for PVH and DSWMH.

**Table 2.** Demographic data of the participants with different severity of white matter hyperintensities (mean (SD)).

	PVH I (n=26)	PVH II (n=32)	PVH III (n=16)	p-value, post hoc
Gender (male:female)	6:20	9:23	4:12	n.s.
Age (years)	74.2 (6.6)	75.4 (6.9)	77.6 (4.9)	n.s.
Education (years)	12.5 (1.7)	12.6 (2.4)	12.3 (1.7)	n.s.
Hypertension	62%	66%	81%	n.s.
Diabetes mellitus	19%	16%	31%	n.s.
Dyslipidemia	38%	59%	44%	n.s.

	DSWMH I (n=14)	DSWMH II (n=27)	DSWMH III (n=33)	p-value, post hoc
Gender (male:female)	2:12	10:17	7:26	n.s.
Age (years)	71.7 (6.2)	75.8 (5.6)	76.8 (6.8)	I vs. III: 0.045
Education (years)	12.4 (1.5)	12.6 (2.1)	12.5 (2.1)	n.s.
Hypertension	29%	81%	70%	I vs. II: 0.003, I vs. III: 0.027
Diabetes mellitus	29%	11%	21%	n.s.
Dyslipidemia	36%	44%	58%	n.s.

PVH: periventricular hyperintensity, DSWMH: deep subcortical white matter hyperintensity, n.s.: not significant.

The relationship between neuropsychological test scores and WMHs are shown in Table 3. There was no significant difference in general cognitive function by the MMSE among three groups for either PVH grade or DSWMH grade before and after age adjustment.

**Table 3.** Performance in neuropsychological tests of the participants with different severity of white matter hyperintensities (mean (SD)).

	PVH I (n=26)	PVH II (n=32)	PVH III (n=16)	p-value (post hoc) without age adjustment			p-value (post hoc) with age adjustment		
				I vs. II	I vs. III	II vs. III	I vs. II	I vs. III	II vs. III
MMSE	27.1 (1.6)	27.2 (1.7)	26.5 (1.6)	1.00	0.737	0.503	1.00	1.00	0.827
Judgement for TMT-A (N:B:A)	62:23:15%	66:9:25%	44:6:50%	n.s.	n.s.	n.s.	-	-	-
Judgement for TMT-B (N:B:A)	31:31:38%	28:13:59%	6:13:81%	n.s.	n.s.	n.s.	-	-	-
Execution time of TMT-A (sec)	51.8 (18.2)	57.1 (30.6)	65.5 (26.2)	1.00	0.329	0.919	1.00	0.596	1.00
Execution time of TMT-B (sec)	133.2 (46.9)	154.4 (61.0)	179.1 (41.1)	0.417	0.026	0.407	0.543	0.07	0.626
Time difference of TMT-B minus TMT-A (sec)	81.3 (46.4)	96.3 (47.8)	113.6 (36.9)	0.671	0.092	0.671	0.81	0.181	0.897

	DSWMH I (n=14)	DSWMH II (n=27)	DSWMH III (n=33)	p-value (post hoc) without age adjustment			p-value (post hoc) with age adjustment		
				I vs. II	I vs. III	II vs. III	I vs. II	I vs. III	II vs. III
MMSE	27.2 (1.3)	27.0 (1.7)	26.9 (1.8)	1.00	1.00	1.00	1.00	1.00	1.00
Judgement for TMT-A (N:B:A)	50:21:29%	67:7:26%	58:15:27%	n.s.	n.s.	n.s.	-	-	-
Judgement for TMT-B (N:B:A)	36:36:28%	22:22:56%	21:9:70%	n.s.	n.s.	n.s.	-	-	-
Execution time of TMT-A (sec)	56.2 (24.6)	58.2 (31.1)	56.4 (22.4)	1.00	1.00	1.00	1.00	1.00	1.00
Execution time of TMT-B (sec)	115.9 (29.4)	156.4 (56.4)	164.4 (55.8)	0.071	0.017	1.00	0.191	0.079	1.00
Time difference of TMT-B minus TMT-A (sec)	59.6 (24.6)	97.0 (46.3)	107.9 (46.7)	0.037	0.003	1.00	0.075	0.011	1.00

N:B:A=Normal:Borderline:Abnormal, n.s.: not significant, values are mean (SD).

As the grade of PVH increased, the rate of “abnormal” clinical judgement increased for the TMT-A and the rate of “normal” decreased and the rate of “abnormal” increased for the TMT-B. However, there were no significant differences in the rate of three clinical judgements among three PVH groups. The testing time required for the TMT-A tended to increase as the PVH grade increased. The testing time required for the TMT-B also showed an increasing trend as the PVH grade increased. Post-hoc analysis showed that there were significant differences in the testing time required for the TMT-B between the PVH grade I and grade III groups without age adjustment (p=0.026). After age adjustment, the difference was not significant, but the testing time required for the TMT-B was marginally longer only for the PVH grade III group compared to the grade I group (p=0.07). The time difference of TMT-B minus TMT-A did not show significant difference among three PVH groups after age adjustment.

Regarding the influence of DSWMH on the TMT subtests, the rate of “normal” clinical judgement for the TMT-B decreased and the rate of “abnormal” increased, but there were no significant differences in the rate of three clinical judgements among three DSWMH groups. The testing time required for the TMT-A was not different among the three DSWMH groups. The testing time required to complete the TMT-B showed an increasing trend as the grade of DSWMH increased. Post-hoc analysis showed significantly longer testing time required for the TMT-B between the DSWMH grade I and grade III groups (p=0.017) without age adjustment. After age adjustment, the testing time required for the TMT-B was marginally longer only for the DSWMH grade III group compared to the grade I group (p=0.079). The time difference of TMT-B minus TMT-A showed an increasing trend as the grade of DSWMH increased. Post-hoc analysis revealed that the time difference was significantly longer for the DSWMH grade II and III groups compared to the grade I group (p=0.037 and p=0.003, respectively) without age adjustment and this difference was still significant after age adjustment between the DSWMH group I and group III (p=0.011).

## Discussion

The present study demonstrated that the severities of both PVH and DSWMH were related to poor performance in the TMT subjects, especially in the TMT-B compared to the TMT-A. Furthermore, isolated executive function assessed by the time difference of TMT-B minus TMT-A was selectively impaired by progression of DSWMH rather than PVH independent of age and general cognitive function.

The finding that the burden of PVH and DSWMH has strong impact on cognitive function in particular attentional and executive function is generally consistent with previous studies. Bolandzadeh *et al.* ^13^ reviewed 14 reports that studied the relationship between WMHs and cognitive function. They reported inconsistent results regarding the distinct influence of PVH and DSWMH on processing speed/executive function. Six ^14^^–^^19^ out of the 14 studies directly compared involvement of PVH and DSWMH in impairment of processing speed/executive function. Two studies ^15^^,^^16^ among them reported stronger impact of PVH compared to DSWMH on processing speed/executive function, but one study ^14^ showed an opposite finding. In addition, three studies ^17^^–^^19^ found no clear difference in the effect of PVH and DSWMH on executive function. Moreover, one different study showed that executive function was more related to DSWMH rather than PVH. ^20^ The exact reason for these conflicting results is not clear. The variability in methods for assessing processing speed/executive function and in clinical characteristics of studied sample might account for the discrepant results. In this study we focused on the distinctive effects of PVH and DSWMH upon the performance in the TMT subtests, which enabled to evaluate the contribution of each type of WMHs to executive function independent of processing speed.

Several studies have revealed the associations of WMHs with isolated components of executive function estimated in the performance of two TMT subtests. Porcu *et al.* ^21^ analyzed the effects of distributed WMHs burden on cognition assessed by the TMT subtests. Significant correlations were obtained between total WMHs burden and scores of the TMT subtests including TMT-B minus TMT-A. They demonstrated that total WMHs burden was correlated with the impairment of all three measures of the TMT subtest scores (i.e. TMT-A, TMT-B, and TMT-B minus TMT-A). The association was the strongest for the TMT-B, followed by TMT-B minus TMT-A, and the TMT-A in this order. Unfortunately, their correlation analysis did not include age as a covariate although age showed the highest correlation coefficient with WMHs burden and all measures of the TMT subtests. Han *et al.* ^22^ also reported a significant correlation between total WMHs volume and the poor performance on TMT-B minus TMT-A and the TMT-A after adjusting age effect. In both reports, however, the distinctive effects of PVH and DSWMH on performance in the TMT subtests were not examined. MacPherson *et al.* ^23^ reported that WMH burden at anterior thalamic radiation and uncinate fasciculus slowed the performance of the TMT-B, but this association was attenuated when controlling the processing speed by other neuropsychological measures. Thus, the current study is the first one that examined the individual influence of PVH and DSWMH on processing speed and executive function separately assessed by the TMT-A, TMT-B, and TMT-B minus TMT-A after adjusting age effect in a clinical population with MCI or without cognitive impairment.

Why does different type of WMHs selectively affect executive function independent of general cognitive function? Respino *et al.* ^24^ demonstrated that disrupted structural connectivity of brain regions caused by WMHs was associated with poor performance in the TMT subtests. The TMT-A score was correlated with connectivity of the supramarginal gyrus, paracentral lobule, thalamus, and pallidum. The TMT-B score was correlated with connectivity of the supramarginal gyrus, pre- and post-central gyri, thalamus, and pallidum. The ratio of TMT-B/A representing isolated set-shifting ability was correlated with the anterior and posterior cingulate gyri, middle frontal cortex, and putamen. Thus, it is plausible that WMHs-related region-specific disrupted structural connectivity may contribute distinctively to behavioral measures of attentional set-shifting and processing speed assessed by the TMT subtests.

Our study has several limitations. In addition to relatively small sample size, the participants were recruited from a single hospital and limited to the subjects who had normal cognitive function or MCI. It will be valuable if the effects of WMHs burden are studied in patient groups including dementia with degenerative or vascular type, which might reveal the relationship between impairment of executive function in dementia and pathological mechanism associated with WMHs. Furthermore, the severity of WMHs were grouped using the Fazekas grading system, but quantitative analysis of their distribution and severity would clarify further the relationship between WMHs and executive function.

In conclusion, exacerbation of PVH and DSWMH differentially affects processing speed and executive function, and DSWMH is more associated with isolated executive functions such as set-shifting and working memory. The TMT subtests may be useful for assessing impairment of isolated executive function in clinical subjects with WMHs.

## Author contributions

Tomiko Nagayama and Shuhei Yamaguchi contributed to the concept and design of the study. Tomiko Nagayama, Sunghoon Yang, and Masao Nagayama were involved in the evaluation of patients. Tomiko Nagayama, Shuhei Yamaguchi and Takuya Nakayama analyzed and interpreted the results. Seiichi Inagaki guided the statistical analyses. Tomiko Nagayama and Shuhei Yamaguchi wrote the manuscript. All authors read and approved the final manuscript.

## ORCID iD

Tomiko Nagayama iD https://orcid.org/0000-0003-3430-8663

Shuhei Yamaguchi iD https://orcid.org/0000-0001-8363-6407

Sunghoon Yang iD https://orcid.org/0000-0003/1655/8060

Masao Nagayama iD https://orcid.org/0000-0002-0332-2027

## Data Availability

Figshare: Effects of white matter hyperintensities on isolated executive function assessed by the Trail Making Test; Underlying data, DOI: https://doi.org/10.6084/m9.figshare.23788503.v3. ^25^ This dataset contains the following underlying data: Data of MMSE and TMT for PVH Data of MMSE and TMT for DSWMH Demographic data of the participants for PVH Demographic data of the participants for DSWMH Data are available under the terms of the Creative Commons Zero “No rights reserved” data waiver (CC BY 4.0 Public domain dedication). Figshare: Effects of white matter hyperintensities on isolated executive function assessed by the Trail Making Test; Extended data, DOI: https://doi.org/10.6084/m9.figshare.23788539.v1. ^26^ This dataset contains the following underlying extended data: PVH and TMT in each gender DSWMH and TMT in each gender Tables of TMT and WMH in each gender Data are available under the terms of the Creative Commons Zero “No rights reserved“ data waiver (CC BY 4.0 Public domain dedication).
