# Supplementary material for: Effects of white matter hyperintensities on isolated executive function assessed by the Trail Making Test
Source: F1000Res. 2023 Aug 22;12:1021. [Version 1] doi: 10.12688/f1000research.139557.1 (PMC10905008; doi:10.12688/f1000research.139557.1)
Supplement: Peer review history

# Reviewer report: Reviewer response for version 1

**DOI:** 10.5256/f1000research.152839.r212610

**Published:** 2024-02-08

**Authors:** Aiko Osawa

**Affiliations:** 1 National Center for Geriatrics and Gerontology, Obu, Japan

This is a very interesting and important study that shows an association between working memory and cerebral white matter lesions in patients seen in a memory clinic.

 In a future paper, we would like to develop this study in the following directions:

- The sample size for each age group may be small and should be increased for further study.
- Patients seen at the memory clinic may be affected not only by white matter lesions, but also by Alzheimer's pathology and other degenerative diseases. Therefore, it would be desirable to conduct a similar analysis on data from healthy older adults and compare the results.

Is the work clearly and accurately presented and does it cite the current literature?

Yes

If applicable, is the statistical analysis and its interpretation appropriate?

Yes

Are all the source data underlying the results available to ensure full reproducibility?

Yes

Is the study design appropriate and is the work technically sound?

Yes

Are the conclusions drawn adequately supported by the results?

Yes

Are sufficient details of methods and analysis provided to allow replication by others?

Yes

Reviewer Expertise:

Neuropsychology

I confirm that I have read this submission and believe that I have an appropriate level of expertise to confirm that it is of an acceptable scientific standard.

# Reviewer report: Reviewer response for version 1

**DOI:** 10.5256/f1000research.152839.r234256

**Published:** 2024-01-12

**Authors:** Tomohide Adachi

**Affiliations:** 1 Saiseikai Central Hospital, Tokyo, Japan

This paper is important in confirming the relationship between cognitive function and cerebral white matter lesions, particularly between frontal lobe functions such as processing speed and executive functions and white matter lesions, divided into PVH and DSWMH. The fact that there was a relationship between the degree of lesions and cognitive decline in DSWMH may help clarify the functional aspects of DSWMH.

 However, as the authors have stated, the number of cases is small, and it isn't easy to accept these results immediately. Furthermore, the composition of the cases targeted is likely to be diverse. In the future, it is considered necessary to conduct research that increases the number of cases and organizes the background of the target group.

Is the work clearly and accurately presented and does it cite the current literature?

Yes

If applicable, is the statistical analysis and its interpretation appropriate?

Yes

Are all the source data underlying the results available to ensure full reproducibility?

Yes

Is the study design appropriate and is the work technically sound?

Yes

Are the conclusions drawn adequately supported by the results?

Yes

Are sufficient details of methods and analysis provided to allow replication by others?

Yes

Reviewer Expertise:

Neurologist, cerebrovascular disease

I confirm that I have read this submission and believe that I have an appropriate level of expertise to confirm that it is of an acceptable scientific standard.
